# The history of oral decitabine/cedazuridine and its potential role in acute myeloid leukemia

**DOI:** 10.1177/20406207231205429

**Published:** 2023-10-17

**Authors:** Robert Briski, Guillermo Garcia-Manero, Hagop Kantarjian, Farhad Ravandi

**Affiliations:** The University of Texas MD Anderson Cancer Center, Houston, TX, USA; The University of Texas MD Anderson Cancer Center, Houston, TX, USA; The University of Texas MD Anderson Cancer Center, Houston, TX, USA; The University of Texas MD Anderson Cancer Center, Unit 428, 1515 Holcombe Boulevard, Houston, TX 77030, USA

**Keywords:** acute myeloid leukemia, 5-azanucleosides decitabine, decitabine/cedazuridine, epigenetics, hypomethylating agents, hypomethylation, novel combination therapy for AML, oral decitabine, oral therapy for AML, therapy for elderly and unfit patients with AML

## Abstract

Decitabine, a member of the 5-azanucleosides, has a dose-dependent mechanism of action *in vitro*: termination of DNA replication at high doses, and inhibition of DNA methyltransferase at low doses. The alteration of DNA methylation patterns by low-dose decitabine is hypothesized to upregulate genes, which promote myeloblast differentiation. In a phase III clinical trial, low-dose decitabine achieved a superior overall response rate (ORR) when compared with ‘treatment choice’ [consisting of low-dose cytarabine (80%) and supportive care (20%)] as a frontline treatment for elderly patients with acute myeloid leukemia (AML). Despite an improved ORR, the median overall survival (OS) for elderly patients with AML was poor, <1 year. In turn, venetoclax was added to low-dose decitabine, the combination of which significantly improved the ORR and median OS in elderly patients with AML. Currently, hypomethylating agents are being combined with other novel therapies as investigational strategies for elderly and unfit patients with AML. They are also being evaluated as components of maintenance therapy in patients achieving remission. An oral formulation of decitabine has been developed which relies on the concomitant use of oral cedazuridine to protect against first pass metabolism. This oral formulation, which has been approved in myelodysplastic syndrome, is intended to increase convenience of use and therefore compliance in patients. This review characterizes the evolution of decitabine, its oral formulation, and its future in the treatment of AML.

## The discovery of decitabine: A story of nucleoside analogs

**Figure 1.**
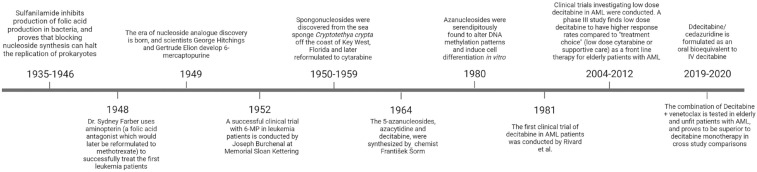
Timeline of decitabine discovery. Events leading to the discovery of decitabine, and the application of decitabine in clinical trials. Source: Created using biorender.com

As will be described below, the desire to discover nucleoside analogs emerged from two important medical discoveries: the discovery of the first sulfa antibiotic (sulfanilamide) and the discovery of the first chemotherapeutic agent (aminopterin). These discoveries demonstrated the importance of nucleoside activity to cell proliferation, and in turn, ignited the race to develop inhibitory nucleoside analogs. As a result of this era of medical discovery, a unique class of nucleoside analogs would emerge called 5-azanucleosides (so named because of a nitrogen atom in the 5′ position of the heterocyclic ring of cytosine) (Figure 1).

In 1935, a time where antibiotics were not readily available, German pathologist Gerhard Domagk found himself in a desperate situation when his daughter contracted streptococcus septicemia. Running with the hypothesis that dyes which preferentially stain bacteria may also kill bacteria without impacting the host,^1^ he treated his daughter with a red dye known as prontosil (a prodrug of sulfanilamide).^2^ Remarkably she recovered, and sulfanilamide became the first widely used antibiotic. Sulfanilamide was used heavily during World War II, seen as a yellow powder applied to wounds of victims, and was even used to treat Winston Churchill’s pneumonia.^3^ Researchers set out to uncover the mechanism of action of this important antibiotic, which actually had nothing to do with its properties as a dye, but rather its ability to block nucleoside synthesis. It was suggested that sulfanilamide blocked thymine synthesis *via* competitive inhibition of para-aminobenzoic acid, the precursor of folic acid in bacteria (folic acid being an important cofactor of the enzyme tetrahydrofolate). To support this hypothesis, researchers demonstrated that when para-aminobenzoic acid,^4^ its downstream metabolite folic acid, or even the end product of the enzymatic reaction, thymine,^5^ were added to bacterial cultures treated with sulfanilamide, these metabolites were able to rescue the cell proliferation capacity of bacteria. Thus, it was shown that sulfanilamide’s ability to impede bacterial replication involved competitive inhibition of para-aminobenzoic acid, which in turn blocked the formation of functional nucleosides.

Blockade of nucleoside development *via* inhibition of folic acid activity proved not only to be an important antimicrobial strategy but also an important antileukemic strategy. In the late 1930s, folic acid was used to treat megaloblastic anemia (a condition which in some ways resembled the bone marrow of leukemia, with accumulation of immature large red cell precursors). Excited about the discovery of this new cofactor, researchers began testing its activity in various cancers. Using a mouse model of breast cancer, researchers suggested folic acid had antitumor activity.^6^ Based on this data, Dr. Sydney Farber decided to treat leukemic patients with folic acid. Though his original report stated that no ‘deleterious effect’ could be seen from the administration of folic acid,^7^ his subsequent publications suggested that folic acid actually induced an ‘acceleration phenomenon’ in leukemic patients.^8,9^ Realizing folic acid potentially fueled leukemia, Dr. Farber tried depriving leukemic cells by using folic acid antagonists (something more in line with the way sulfa drugs induce bacterial cell death). Using aminopterin (a folic acid inhibitor which would later be reformulated to methotrexate), Farber achieved a response rate of 62% (*n* = 16) in his leukemic patients^9^ and was gifted the name ‘father of chemotherapy’.

By 1947, inhibition of nucleoside activity was at the cornerstone of drug discovery for rapidly proliferating diseases. Armed with the knowledge of competitive inhibition, researchers decided to develop a new class of drugs called nucleoside analogs: molecules, which looked similar to nucleosides but lacked their functional capacity. Such molecules could preferentially be taken up by rapidly dividing cells and induce cell death.

**Figure 2.**
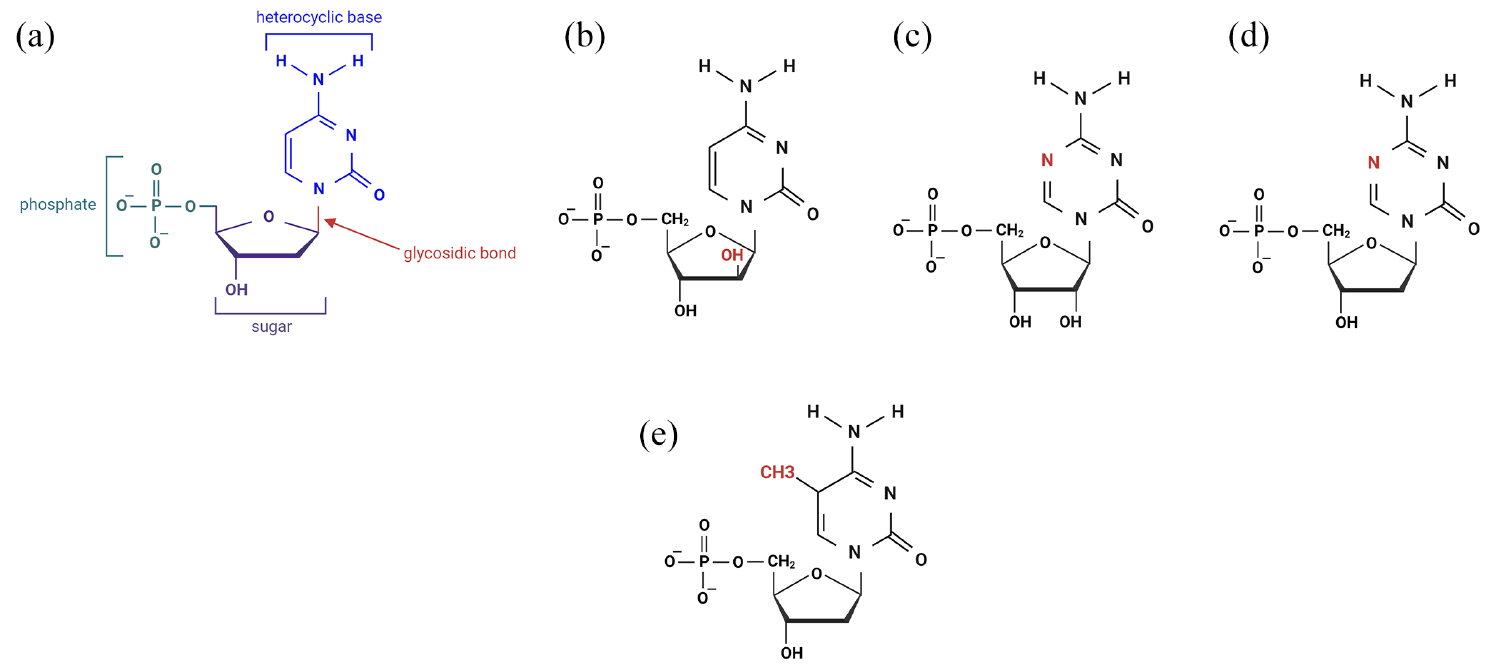
(a) The chemical structure of the DNA nucleoside deoxycytidine (there are four potential locations for chemical modification: the phosphate group, the sugar, the glycosidic bond, and the heterocyclic base). (b) The chemical structure of cytarabine (it differs from the RNA nucleoside cytidine by the conformation of the 2′ hydroxyl group in the sugar). (c) Azacytidine (differs from cytidine by the presence of a nitrogen atom in the 5′ position of the heterocyclic ring). (d) Decitabine (differs from deoxycytidine by the presence of a nitrogen atom in the 5′ position of the heterocyclic ring). (e) Methylated deoxycytidine. Source: Created using biorender.com

Nucleosides have four main components, and therefore four targets of drug modification: a heterocyclic base, a glycosidic bond, a sugar base and a phosphate group (Figure 2).^10^ In 1947, American scientists George Hitchings and Gertrude Elion set out to develop as many synthetic non-functional analogs of nucleosides as possible. While nucleoside analogs did not prove to be useful antibiotics, they did become significant chemotherapeutic agents, antiviral therapies (with drugs like acyclovir), immunosuppressants (such as azathioprine, an important drug used in transplant), and even a treatment for gout (with xanthine oxidase inhibitors like allopurinol). The research by Hitchings *et al.* gave rise to a very important chemotherapeutic drug, 6-mercaptopurine (6-MP).^11^ After demonstrating activity of 6-MP in leukemic mouse models, this drug was tested in leukemic patients at Memorial Sloan Kettering in 1952.^12^ Given its success compared with methotrexate and steroids in acute leukemia, 6-MP became FDA approved in 1953. With a newfound interest for nucleoside analogs to treat cancer, researchers searched high and low, until their journey took them to the depths of the Caribbean Sea off the coast of the Florida Keys where they found a very important bioactive compound from the sea sponge *Cryptotethya crypta*. It had been known that sponges utilize chemicals to fight off their predators, thus are important reservoirs of bioactive compounds. The bioactive compound extracted from *C. crypta* was reformulated into the antileukemic drug cytosine arabinoside (also known as cytarabine or Ara-C).^13^ This nucleoside analog looks almost identical to cytidine (the RNA nucleoside) with one key difference, the hydroxyl group in the 2′ position of the sugar has a different conformation (a so called epimer) (Figure 2). This slight conformational difference is the reason for cytarabine’s extremely potent activity in acute myeloid leukemias (AML).

Looking for other novel nucleoside analogs, Czechoslovakian chemist František Šorm began focusing on azanucleosides – cytosine analogs with a nitrogen atom incorporated into the heterocyclic ring. Azanucleosides were felt to be similar to cytosine: with similar molecular weights, no additional functional groups and an almost isosteric conformation.^14^ Hoping azanucleosides would be different enough to throw off the structure of RNA and DNA, Šorm synthesized a specific group of azanucleosides called the 5-azanucleosides: 5-azacytidine (azacytidine) and 5-aza-2′-deoxycytidine (decitabine) (so named because of the incorporation of a nitrogen atom at the 5′ position of the heterocyclic ring, Figure 2). Decitabine was shown to have antileukemic activity in mice,^15^ and eventually was evaluated in clinical trials as a competitor of cytarabine (see section below ‘Use of decitabine in myeloid leukemias: A preference for lower doses’).

## Cytosine methylation as an epigenetic modifier

For years, embryologists struggled to understand how totipotent eukaryotic stem cells could differentiate into diverse cells when all differentiated progeny retained the exact same set of genes. For instance, the genes of a myocardiocyte can be found within a retinal cell and vice versa. If portions of DNA were not deleted during differentiation, something clearly regulated the way in which DNA was read to give rise to differential gene expression. The first major break though in transcriptional regulation came with the discovery of cytosine methylation (a discovery which would give birth to the field of epigenetics).

By the 1940s, it was known that DNA consisted of four nucleosides: adenine, thymine, guanine, and cytosine. Each nucleoside could be separated on filter paper by liquid chromatography, then visualized with ultraviolet (UV) spectrophotometry based on individual molecular absorption patterns. As experimentation continued, the presence of a fifth nucleoside was revealed.^16^ This nucleoside could be seen adjacent to cytosine on the spectrophotometry graph and was termed ‘epicytosine’. Epicytosine was later identified to be cytosine with a methyl group at the fifth position 5-methylcytosine (5mC). Researchers discovered that the concentration of 5mC varied by tissue within an organism.^17^ Eventually, it was hypothesized that the differential tissue expression of 5mC meant that 5mC was responsible for transcriptional regulation and cell differentiation.^18–22^ Only by sure serendipity would the discovery of decitabine later support the hypothesis that 5mC regulates gene expression (see section below).

## Azanucleosides as the first epigenetic modifying drugs: A serendipitous story of a dose-dependent mechanism of action

By the 1970s, azanucleosides were among three classes of chemotherapeutic agents used to treat cancer patients: antimetabolites (the class to which azanucleosides belong), alkylating agents, and anti-tumor antibiotics. Though these drugs were capable of inducing long-term remissions in select patients, clinicians began noticing secondary cancers among survivors. This led researchers to question whether cancer patients had a genetic predisposition to additional primary malignancies, or if chemotherapeutic agents themselves were carcinogenic. Several *in vivo* and *in vitro* studies were conducted to investigate the mutagenicity of chemotherapeutic agents.^23,24^ One prevailing *in vitro* model at the time was the use of hamster or mouse embryonic stem cells. These embryonic cells were slow growing with low rates of differentiation. As such, they were quiescent, less prone to enter S-phase, and were able to survive treatment. After incubation in chemotherapeutic solution, cells were injected into mice to see if they would transform from normal embryonic cells into cancerous cells, thereby confirming mutagenicity.^25^ Following investigations with decitabine, scientists discovered something unexpected, rather than die or transform into neoplastic cells, embryonic cells were stimulated to differentiate into myocytes *in vitro*.^26,27^

Knowing that unique 5mC methylation patterns correlated with different cell types, Jones and Taylor^22^ hypothesized that decitabine might induce differentiation by blocking methylation of cytosine at the 5′ position. In their experimentation, they replicated the finding that low-dose decitabine induces differentiation of embryonic cells into myocytes, and further showed that this correlated with a reduction in DNA methylation as determined by high-performance liquid chromatography with UV spectroscopy and by restriction enzyme studies (whereby restriction enzymes from bacteria, which normally cleave unmethylated DNA, can be used to qualitatively demonstrate unmethylated DNA in the form of extra bands in agarose gel). As the dose of decitabine was increased, however, the number of myocytes declined, suggesting that the drug was playing more of a cytotoxic effect at higher doses. This led to the hypothesis that decitabine has a dose-dependent mechanism of action: it acts as a hypomethylating agent at low doses, and a cytostatic agent which terminates DNA replication at high doses.

An important finding in the study by Jones and Taylor was the discordance between low levels of decitabine incorporation into DNA and the high degree of hypomethylation it could cause. Only 5% of decitabine incorporation into DNA resulted in an 85% reduction of DNA methylation.^22^ This suggested that decitabine blocked DNA methylation through a mechanism other than acting as a functional cytosine analog, which lacks the ability to be methylated. It was later shown that the nitrogen at the 5′ position of the heterocyclic ring of 5-azanucleosides induces a nucleophilic attack against DNA methyltransferase (DNMT), thereby forming a covalent bond and rendering DNMT inactive.^28^ With enough DNMT inhibited, DNA synthesis could proceed without methylation of cytosine, allowing for transcription of genes, which could drive cell differentiation.

From here, other experiments were conducted to study the ability of decitabine to induce differential gene expression and cell differentiation. Low-dose decitabine was able to induce reactivation of inactive X chromosomes in humanized mouse cells.^29^ Decitabine was also shown to induce fetal hemoglobin expression in patients with sickle cell anemia at 0.15 mg/kg/day.^30^ This was felt to be through the same mechanism as azacytidine, which was shown to demethylate (therefore allow transcription of) the fetal hemoglobin gene.^31^ A study by Pinto *et al.*^32^ in 1984 also suggested that monoblastic cells from patients with AML could be differentiated into macrophages following treatment with 1 μM/L of decitabine. This was performed by incubating leukemia cells with decitabine. Following treatment, cells were observed to undergo morphologic changes and acquire the ability to phagocytose latex particles, suggesting that they transformed from myelomonoblasts to macrophages.

## Use of decitabine in myeloid leukemias: A preference for lower doses

The first clinical trial of decitabine took place in Quebec in 1981.^33^ As a phase I study, Rivard *et al.* investigated different dosing schedules in pediatric patients with relapsed and refractory acute lymphoblastic leukemia (ALL) and AML. They wanted to determine maximally tolerated doses of decitabine, as well as the optimal dose for antileukemic activity. Although it had been suggested the starting dose for antileukemic drugs in phase I trials should be 1/3 the lethal dose in mice,^34,35^ Rivard *et al.* started at 1/10th the lethal dose in mice (0.75 mg/kg administered over 12 h^33, 36^). The dose and rates of infusion were increased incrementally until post infusion serum studies from patients reached optimal antileukemic activity (whereby patient serum containing decitabine was incubated with a leukemic cell line and assessed for apoptotic activity). The highest dose and infusion rate achieved was 80 mg/kg infused over 40 h. Rivard *et al.* found that while lower doses of decitabine (0.75–17 mg/kg, given continuously at a rate of 1 mg/kg/h) were capable of reducing the leukemic burden (seen with a reduction in circulating blasts, clearance of a malignant pleural effusion in a patient, reduced bone pain and a reduction in testicular size), marrow responses were not seen, and remissions were short lived (3–12 days). Improved and more prolonged responses were seen in patients receiving doses ⩾24 mg/kg (infused at a rate 1 mg/kg/h continuously), including some marrow responses starting at 36 mg/kg and meningeal responses at 44 mg/kg. Response duration for doses ⩾24 mg/kg ranged from 29 to >50 days. Unfortunately, higher doses of decitabine resulted in more prolonged myelosuppression. This was shown to be the result of activity against normal hematopoietic elements: among the patients in the trial, three patients without marrow disease received either 36 or 80 mg/m^2^ within 2 days, resulting in severe neutropenia (<100 cells/μL) and thrombocytopenia (<10,000 platelets/μL) lasting an average of 24 and 42 days, respectively. As investigations of decitabine continued, the focused remained on higher cumulative doses per day (Table 1). Unfortunately, due to the focus on maximally tolerated doses, further trials were placed on hold due to concerns of myelotoxicity and the investigation of decitabine would only continue in myelodysplastic syndrome (MDS) and chronic myeloid leukemia (CML) for the next several years.

**Table 1.** Clinical trials investigating higher daily doses of decitabine in AML, including trials with combination therapy.

Study	No. of points	Cumulative decitabine dose	Rate of infusion	Response %
Salvage therapy				
Rivard *et al*.^33^	9	36–80 mg/kg	36–44 h*	33
Momparler *et al*.^37^	6	45–100 mg/kg	40–90 h*	89
Richel *et al*.^38^	16	250–1000 mg/m^2^ (± amsacrine)	6h/6 d	62
Willemze *et al*.^39^	22	250 mg/m^2^ (+ amsacrine or idarubicin)	6h / 6d	68
Willemze *et al*.^40^	63	250 mg/m^2^ (+ amsacrine or idarubicin)	6h / 6d	39
Front-line therapy				
Petti *et al*.^41^	12	90–120 mg/m^2^	4h / 3d	33
Schwartsmann *et al*.^42^	8	90 mg/m^2^ (+ daunorubicin)	4h / 5d	75

* Continuous infusion.

AML, acute myeloid leukemia; d, days; h, hours.

In an extension to their phase I study, Momparler *et al.* investigated the hypomethylating potential of decitabine in patients with acute leukemia. They extracted serum samples from two patients, one with AML and one with ALL, at different time points in order to quantitate the degree of methylation before and after decitabine infusion.^43^ The dose administered for the patient with ALL was 24 mg/kg, and the dose administered for the patient with AML was 40 mg/kg (in both patients, decitabine was administered 1 mg/kg/h continuously). Their investigational technique involved extracting white cells from peripheral blood of patients *via* centrifugation and Ficoll-Paque, measuring the amount of 5mC present in the white cell extract, and incubating the white cells with radiolabeled cytosine to evaluate replicative potential (increased radiolabeled cytosine incorporation into DNA was suggestive of more time spent in S-phase and higher replicative potential). They reported an 80% reduction in methylation post-decitabine infusion (as suggested by the concentration of 5mC), and a reduction in replicative potential (as suggested by a reduction in radiolabeled cytosine incorporation post decitabine infusion). With these findings, they felt decitabine acted as a hypomethylating agent and was able to induce differentiation. The only caveat to their experimental technique was that they did not prove the white cell extract was purely leukemic blasts or differentiated progeny of leukemic blasts.

Because it was suggested that decitabine may act as a hypomethylating agent at doses of 40 mg/m^2^ in AML patients, researchers began looking at low-dose decitabine as a means to treat patients with advanced MDS [MDS with excess blasts, and refractory anemia with excess blasts (RAEB) in transformation (based on the FAB classification scheme defined as >20% bone marrow blasts, >4% peripheral blasts, or numerous auer rods within dysplastic cells)], a disease which was felt to be the result of hypermethylated DNA. In a phase I trial in advanced MDS, patients were treated with either 45 or 50 mg/m^2^/day for three consecutive days.^44^ In total, 7/10 patients experienced a hematologic response, and 5/10 patients experienced a complete remission (CR) or partial remission (PR) (defined as reduction in number of blasts). In this study, it was not shown unequivocally that the disease modifying activity of decitabine (at this dose) was due to its hypomethylating, opposed to its cytostatic properties. These results were further validated in a larger phase II trial.^45^

As AML therapies continued to improve, there remained a population which was particularly challenging to treat, the elderly and unfit. Due to their lack of physiologic reserve, these patients were at a high risk of treatment-related mortality.^46,47^ This led to trials investigating the potential role of decitabine in patients with AML.

Proposing an even more cautious dosing schedule than used in MDS, Issa *et al.*^48^ treated patients with 10–20 mg/m^2^/day IV decitabine for 10 days [though the total cumulative dose was similar to MDS (150 mg/m^2^/cycle) decitabine was spread out over 10 days instead of 3 days].^48^ Of the 37 patients treated, 22% experienced a response. The duration of response and median overall survival (OS) were not reported (Table 2). Hoping to prove low-dose decitabine functions as a hypomethylating agent in AML, Issa *et al.* investigated the methylation status of an important cell cycle regulator, *p15* (which when methylated, becomes inactive allowing the cell to progress through the cell cycle), before and after decitabine infusions. They found no differences in the level of hypomethylation of *p15* before or after treatment with decitabine, which as suggested by their article, may have been due to a flaw in experimental design (they did not ensure the cells being analyzed were leukemic blasts or differentiated progeny of leukemic blasts).

**Table 2.** Clinical trials investigating low-dose decitabine in AML, including trials with combination therapy.

Study	No. of points	Cumulative decitabine dose	Rate of infusion	Therapy status	Response (%)	Median OS
Issa *et al*.^48^	37	100–200 mg/	1 h/10 d	Salvage	22	NR*
Cashen *et al*.^49^	55	100 mg/m^2^	1 h/5 d	Frontline	25	7.7 mo
Kantarjian *et al*.^50^	485	100 mg/m^2^	1 h/5 d	Frontline	17.8	7.7 mo
Ritchie *et al*.^51^	52	200 mg/m^2^	1 h/10 d	Frontline	40	8.7 mo
Blum *et al*.^52^	53	200 mg/m^2^	1 h/10 d	Frontline	64	12.7 mo
DiNardo *et al. de novo* AML^53^	70	200 mg/m^2^ + venetoclax 400 mg daily	1 h/10 d	Frontline	89	18.1 mo
DiNardo *et al.* untreated secondary AML^53^	15	200 mg/m^2^ + venetoclax 400 mg daily	1 h/10 d	Frontline	80	7.8 mo
DiNardo *et al.* treated** secondary AML^53^	28	200 mg/m^2^ + venetoclax 400 mg daily	1 h/10 d	Frontline	61	6 mo
DiNardo *et al.* AML^53^	55	200 mg/m^2^ + venetoclax 400 mg daily	1 h/10 d	Salvage	62	7.8 mo
Yilmaz *et al.* mutFLT-3 AML^54^	23	200 mg/m^2^ + venetoclax 400 mg daily + quizartinib 30–40 mg daily	1 h/10 d	Salvage	78	7.6 mo

* Not reported.

** Patients who received treatment for their antecedent hematologic disorder.

AML, acute myeloid leukemia; d, days; h, hour; m, months.

Investigations of low-dose decitabine in AML proceeded to a phase II trial in 53 elderly patients with untreated AML. The overall response rate (ORR) was 26% and the median OS was 7.7 months.^49^ Due to the positive signals in preceding trials, investigations of decitabine advanced to a large phase III randomized study in elderly patients with untreated AML.^50^ Low-dose decitabine (20 mg/m^2^ IV for 5 days every 4 weeks) was compared with treatment of choice [this consisted of 80% of patients receiving low-dose cytarabine (20 mg/m^2^ subcutaneously for 10 days) and 20% receiving supportive care]. Although the median OS was not statistically significant between the two groups, the ORR rate was higher among patients receiving low-dose decitabine (17% *versus* 7.8%, *p* = 0.001). With longer follow-up, there was a significant superior OS for patients treated with decitabine.

Subsequent studies suggested that longer exposure times of low-dose decitabine (i.e. 10 days per cycle instead of 5 days) can produce even better ORR (40–64%).^51,52^ Given its superior response rate compared to low-dose cytarabine, low-dose decitabine became a major player in the treatment of unfit and elderly patients with AML.

## Development of oral decitabine

As described in the trials above, IV decitabine is administered daily for 5–10 days at an infusion center every month. The administration of IV decitabine for 5–10 days per cycle at an infusion center can be particularly cumbersome for elderly and unfit patients. Logistically speaking, 1 out of every 4 weeks would be spent with the following schedule: drive to an infusion center, wait for therapy to be prepared, receive an infusion, and drive back home (each day could take up to 5 h or more).^55^ Moreover, this will occur during the part of the cycle where patients feel most fit. Patients will already spend the subsequent weeks of their cycle with fatigue from therapy related cytopenia, receiving frequent lab checks, and possibly sitting in the infusion center for hours receiving blood products. This can be a grueling schedule for those receiving therapy for 1 year or more. Unfortunately, due to the logistical constraints of therapy, some patients elect to forgo additional treatment and pursue hospice to focus on quality of life measures. In order to spend more quality time at home, patients would prefer their treatment in the form of a pill. Such a formulation might also improve patient compliance. Thus, researchers began searching for an oral preparation of decitabine.

The initial phases of oral decitabine development were plagued by major variations in the serum concentration of decitabine. In the phase I study of oral decitabine, serum levels among 12 patients ranged from 4% to 14%.^56^ The differences in bioavailability among patients was hypothesized to be the result of decitabine’s metabolism by cytidine deaminase (CDA), an enzyme with high expression levels in the gut and liver.^57,58^ Unlike an IV infusion, which is typically administered to a vein above the diaphragm and enters the superior vena cava, thereby skipping first pass metabolism, oral decitabine must pass through the gut and liver before entering arterial circulation. In turn, oral decitabine has more potential to be degraded by CDA than IV decitabine before reaching leukemic cells in the marrow.

**Figure 3.**
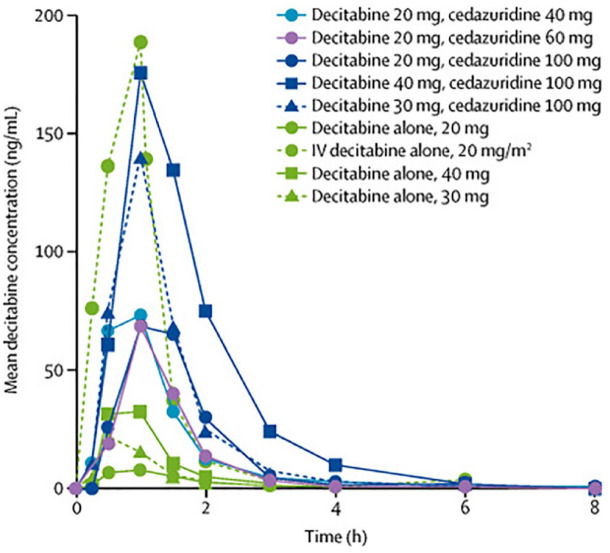
Patient serum decitabine concentration by time following infusion. Area under the curve for the combination of oral decitabine/cedazuridine *versus* oral decitabine alone *versus* IV decitabine. The dose of decitabine/cedazuridine 35 mg/100 mg was not directly tested. However, based on the areas under the curve for decitabine/cedazuridine 40 mg/100 mg and 30 mg/100 mg, it was determined that 35 mg/100 mg would be the most comparable to 20 mg decitabine IV. Source: Reprinted from Savona *et al*.,^58^ with permission from Elsevier.

Researchers began looking for inhibitors of CDA, which they could co-administer with decitabine in order to protect decitabine from first pass metabolism. Eventually they discovered cedazuridine, which could be co-administered as an oral therapy. In phase I and phase II pharmacokinetic studies, it was determined that the most accurate dosing of oral decitabine/cedazuridine was 35 mg/100 mg, which would give the same area under the curve of decitabine as IV decitabine 20 mg (Figure 3).^58,59^ This dose was further validated with pharmacodynamic testing utilizing global hypomethylation assays, which suggested IV decitabine 20 mg and oral decitabine/cedazuridine 35 mg/100 mg are bioequivalent doses.^59^

## Combination strategies with decitabine: Regimens which could be entirely oral

Since the median OS for patients with AML receiving low-dose decitabine monotherapy was poor, <1 year, researchers began investigating decitabine-based combinatorial strategies. The first combination investigated was low-dose decitabine with venetoclax, an inhibitor of the B-cell lymphoma 2 protein (BCL-2). BCL-2, an oncogene, received its name following its discovery in follicular lymphoma whereby the translocation of chromosomes 14 and 18 leads to its overexpression.^60,61^ Although BCL-2 inhibitors were initially developed for lymphoid malignancies, mRNA expression studies in mice incidentally demonstrated that BCL-2 is also upregulated in early myeloid progenitor cells of the bone marrow.^62^ Naturally, investigations of BCL-2 inhibition spilled over into the field of AML. In early preclinical models, BCL-2 inhibitors were found to work synergistically with azanucleosides to induce myeloblast cell death.^63^ As a result of this positive signal, the combination of venetoclax with low-dose azanucleoside (either azacytidine or decitabine) proceeded to the first clinical trial in 2019.^64^ The ORR looked promising (60–73%), and the optimal dose of venetoclax was determined to be 400 mg daily when given with IV decitabine (20 mg/m^2^ for 5 days) or IV azacytidine (75 mg/m^2^ for 7 days). The phase I study determined the toxicity of this combination to be acceptable, and investigations proceeded to a phase II study called the DEC10-VEN. Decitabine for 10 days plus venetoclax (DEC10-VEN) investigated IV decitabine 20 mg/m^2^ for 10 days per each 28 days cycle combined with continuous daily venetoclax (400 mg) in four different arms: untreated *de novo* AML (*n* = 70), untreated secondary AML (*n* = 15), previously treated secondary AML (specifically, patients who received therapy for their antecedent hematologic disorder, *n* = 28) and relapsed/refractory AML (*n* = 55).^53^ The most impressive outcome was reported for patients with *de novo* untreated AML, who experienced an ORR of 89% and a median OS of 18.1 months (the remainder of outcomes can be seen in Table 2). Although no direct head to head comparisons were made between low-dose decitabine monotherapy and the combination of decitabine and venetoclax, it can be inferred from cross study comparisons that the combination produces a superior ORR and OS.^50,53,65^

In select patients, the combination of IV decitabine and venetoclax has proven so effective, investigators are beginning to assess the possibility of stopping therapy for a treatment free remission (TFR). In one small study, the reported median OS for patients who completed ⩾12 months of therapy (consisting of venetoclax plus decitabine/azacytidine/or low-dose Ara-C) and achieve a CR (duration of CR was not specified) was an impressive 71.3 months.^66^ Given this outstanding OS, investigators proceeded to hold therapy for 13 patients after completing ⩾12 months of therapy and achieving a CR. The median OS of this population was still 71.3 months, and the median TFR was 45.8 months. It is important to note that the TFR benefit was most substantial in those with an *IDH2* or *NPM1* mutation who achieved an MRD negative CR as determined by flow cytometry (six of the seven patients still in TFR at the time of data cutoff fit this description). Due to the success of IV decitabine with venetoclax in select patients, researchers are now investigating the combination of decitabine/cedazuridine with venetoclax as an entirely oral regimen (Table 3).

**Table 3.** Ongoing clinical trials investigating low-dose decitabine-based combination therapy for AML: regimens, which could be entirely oral.

Study	Disease setting	Clinical trial no.
Oral decitabine/cedazuridine + venetoclax	Frontline or relapsed/refractory	NCT04746235
Oral decitabine/cedazuridine + venetoclax	High ELN risk patients unexposed to venetoclax and have received ⩽1 cycle of DNMTi	NCT04817241
Decitabine + midostaurin	Frontline in unfit patients	NCT04097470
Decitabine + pacritinib	Frontline in elderly fit patients	NCT02532010
Very low-dose decitabine + venetoclax	Unfit patients without prior HMA or venetoclax exposure	NCT05184842
Decitabine + talazoparib*	Frontline or as first salvage therapy	NCT02878785
KRT-232** ± decitabine or low-dose Ara-C	Relapsed/refractory or frontline AML secondary to MPN	NCT04113616
Oral decitabine/cedazuridine + SNDX-5613*** + venetoclax	Relapsed/refractory	NCT05360160
Oral decitabine/cedazuridine + entrectinib	Relapsed/refractory TP53 mutated	NCT05396859
Oral decitabine/cedazuridine + venetoclax + gilteritinib	Frontline or relapsed/refractory FLT3 mutated	NCT05010122
Oral decitabine/cedazuridine + venetoclax + ivosidenib or enasidenib	Relapsed/refractory with *IDH1* or *IDH2* mutations	NCT04774393
Decitabine + defactinib	Fit patients, frontline or relapsed/refractory, having received no more than one prior HMA and no prior SCT	NCT05636514
Maintenance oral decitabine/cedazuridine ± venetoclax, gilteritinib, enasidenib, or ivosidenib	In first complete remission (CR/CRi)	NCT05010772

* This strategy should be considered with caution given the recent concern for PARP inhibitor related myeloid neoplasms.^67,68^

** Oral inhibitor of MDM2 (murine double minute 2).

*** Oral menin inhibitor.

AML, acute myeloid leukemia; CRi, complete remission with incomplete count recovery; DNMTi, DNA methyltransferase inhibitor; ELN, European LeukemiaNet; HMA, hypomethylating agent; MPN, myeloproliferative neoplasms; PARP, poly-ADP ribose polymerase; SCT, stem cell transplant.

The combination of decitabine and venetoclax does not seem to be as effective in patients with *mutTP53*. In a *post hoc* analysis of the DEC10-VEN study, patients with *mutTP53* (*n* = 35) experienced an inferior ORR and median OS when compared with *wtTP53* (*n* = 83): 66% *versus* 89% (*p* = 0.002) and 5.2 months *versus* 19.4 months (*p* < 0.0001), respectively.^69^ Among patients with *mutTP53*, those with multiple *TP53* mutations were more likely to relapse or have refractory disease [multiple *TP53* mutations were noted in 4 of 7 (57%) responding patients without a relapse *versus* 13 of 16 (81%) who had a relapse after a response *versus* 10 of 10 (100%) who had refractory AML (*p* = 0.049)]. However, the median OS was not statistically different between the *mutTP53* and *wtTP53* groups. The variant of allelic frequency (VAF) of *mutTP53* did not seem to have much prognostic significance (that being said, the majority of patients in the DEC10-VEN study had a VAF <50%. As suggested by Bernard *et al*.,^70^ VAF’s >50% and multiple *TP53* mutations are more specific surrogates of biallelic *mutTP53*. The significance of biallelic *mutTP53* is that 100% of the proapoptotic p53 protein is mutated; thus, these cells are more resistant to apoptosis). Of those with *mutTP53* who responded, there was a remarkable reduction in *mutTP53* VAF (median reduction of 28.5%). For those who were refractory, there was no significant change in *mutTP53* VAF. Finally, this *post hoc* analysis attempted to compare the 35 *mutTP53* patients receiving DEC10-VEN with 17 *mutTP53* patients receiving DEC10 monotherapy on a different clinical trial. One must take into consideration the degree of potential confounding with respect to patient characteristics and molecular profiles in such a small sample. But in their evaluation, they did not find the addition of venetoclax to confer a significantly improved ORR or OS in patients with *mutTP53*.

With proof of concept that outstanding remissions can be achieved in select AML patients receiving low-intensity therapy, investigators are now looking to combine oral decitabine/cedazuridine with other novel agents such as isosorbide dehydrogenase (IDH)1/2 inhibitors, fms-like tyrosine kinase 3 (FLT3) inhibitors, menin inhibitors, or other agents, with or without the use of venetoclax to improve outcomes for all AML patients. Additionally, there are studies looking at the use of maintenance oral decitabine for patients who have achieved a CR. Currently, there are 13 trials of oral decitabine/cedazuridine containing regimens under investigation, all of which could be entirely oral (Table 3).

Of these strategies, an analogous phase I/II study has been completed for the combination of low-dose IV decitabine, venetoclax, and quizartinib in patients with relapsed/refractory *FLT3* mutated AML (Table 2).^54^ The ORR was 78% with a median OS of 7.6 months. Similar results were reported with other FLT3 inhibitors used off label in a small subgroup of patients with relapsed/refractory *FLT3* mutated AML in the DEC10-VEN trial.^53^ They included 10 patients who received sorafenib (*n* = 5), gilteritinib (*n* = 4), and midostaurin (*n* = 1). The ORR for these patients was 86%.

## Conclusion

Following the discovery of the dose-dependent mechanism of action of decitabine (a drug which serves as a cytostatic inhibitory nucleoside analog at high doses, and as a DNMT inhibitor with the potential to alter DNA methylation patterns at low doses), low-dose decitabine has become an important tool in the treatment of AML. Low-dose decitabine works synergistically with venetoclax in AML, creating a well-tolerated therapy with the potential to induce a median OS of 71.3 months in select patients. Unfortunately for many patients, such an OS has not been achieved owing to genetic diversity of AML: amongst all untreated *de novo* AML patients, the median OS is 18 months. In an effort to extend a durable long-lasting survival to all AML patients, researchers are looking to combine low-dose decitabine with other novel agents with or without the use of venetoclax. Because IV decitabine infusions are cumbersome to receive and may impact the quality of life for many patients, scientists developed an oral formulation of decitabine (which relies on the CDA inhibitor cedazuridine to bypass first pass metabolism). The oral formulation of decitabine appears to have the same pharmacokinetic and pharmacodynamic properties as IV decitabine as determined by studies in MDS. In the current era of AML, investigators are evaluating the possible use of well-tolerated oral regimens, which patients can take at home, avoiding the need for frequent visits to the clinic.

## Declarations

**Ethics approval and consent to participate:** This is a review article of information freely available in the public domain (i.e. a review of work already published by others), thus does not require ethics approval or informed consent.

**Consent for publication:** This is a review article of information freely available in the public domain (i.e. a review of work already published by others), thus does not require informed consent.

**Availability of data and materials:** Not applicable.

## References

[1] Bickel MH . The development of sulfonamides (1932–1938) as a focal point in the history of chemotherapy. Gesnerus 1988; 45: 67–86. 3042521

[2] Domagk G . Chemotherapie der Streptokokken-Infektionen. Klinische Wochenschrift 1936; 15: 1585–1590.

[3] Lesch JE . The first miracle drugs: how the sulfa drugs transformed medicine. New York, NY: Oxford University Press, 2007.

[4] Teply LJ Axelrod AE Elvehjem CA . Sulfapyridine bacteriostasis of *Lactobacillus arabinosus* and its counteraction. J Pharmacol Exp Ther 1943; 77: 207.

[5] Lampen JO Jones MJ . The antagonism of sulfonamides by pteroylglutamic acid and related compounds. J Biol Chem 1946; 164: 485–486. 20989508

[6] Lewisohn R Leuchtenberger C Leuchtenberger R , et al. The influence of liver *L. casei* factor on spontaneous breast cancer in mice. Science 1946; 104: 436–437. 10.1126/science.104.2706.436 17819687

[7] Farber S Cutler EC Hawkins JW , et al. The action of pteroylglutamic conjugates on man. Science 1947; 106: 619–621. 17831847 10.1126/science.106.2764.619

[8] Farber S . Some observations on the effect of folic acid antagonists on acute leukemia and other forms of incurable cancer. Blood 1949; 4: 160–167. 18107667

[9] Farber S Diamond LK Mercer RD , et al. Temporary remissions in acute leukemia in children produced by folic acid antagonist, 4-aminopteroyl-glutamic acid (aminopterin). N Engl J Med 1948; 238: 787–793. 18860765 10.1056/NEJM194806032382301

[10] Seley-Radtke KL Yates MK . The evolution of nucleoside analogue antivirals: a review for chemists and non-chemists. Part 1: Early structural modifications to the nucleoside scaffold. Antiviral Res 2018; 154: 66–86. 29649496 10.1016/j.antiviral.2018.04.004 PMC6396324

[11] Elion GB . The purine path to chemotherapy. Science 1989; 244: 41–47. 2649979 10.1126/science.2649979

[12] Burchenal JH Murphy ML Ellison RR , et al. Clinical evaluation of a new antimetabolite, 6-mercaptopurine, in the treatment of leukemia and allied diseases. Blood 1953; 8: 965–999. 13105700

[13] Lichtman MA . A historical perspective on the development of the cytarabine (7 days) and daunorubicin (3 days) treatment regimen for acute myelogenous leukemia: 2013 the 40th anniversary of 7+3. Blood Cells Mol Dis 2013; 50: 119–130. 23154039 10.1016/j.bcmd.2012.10.005

[14] Gut J . Aza analogs of pyrimidine and purine bases of nucleic acids. In: Katritzky AR (ed.) Advances in heterocyclic chemistry. London, UK: Academic Press, 1963, pp. 189–251. 10.1016/s0065-2725(08)60526-7 14087220

[15] Sorm F Pískala A Cihák A , et al. 5-Azacytidine, a new, highly effective cancerostatic. Experientia 1964; 20: 202–203. 10.1007/BF02135399 5322617

[16] Hotchkiss RD . The quantitative separation of purines, pyrimidines, and nucleosides by paper chromatography. J Biol Chem 1948; 175: 315–332. 18873306

[17] Sheid B Srinivasan PR Borek E . Deoxyribonucleic acid methylase of mammalian tissues. Biochemistry 1968; 7: 280–285. 4991479 10.1021/bi00841a034

[18] Scarano E . The control of gene function in cell differentiation and in embryogenesis. Adv Cytopharmacol 1971; 1: 13–24. 5163242

[bibr19-20406207231205429] Comings DE . Methylation of euchromatic and heterochromatic DNA. Exp Cell Res 1972; 74: 383–390. 4673149 10.1016/0014-4827(72)90391-6

[bibr20-20406207231205429] Holliday R Pugh JE . DNA modification mechanisms and gene activity during development. Science 1975; 187: 226–232. 1111098

[bibr21-20406207231205429] Riggs AD . X inactivation, differentiation, and DNA methylation. Cytogenet Cell Genet 2008; 14: 9–25. 10.1159/000130315 1093816

[22] Jones PA Taylor SM . Cellular differentiation, cytidine analogs and DNA methylation. Cell 1980; 20: 85–93. 6156004 10.1016/0092-8674(80)90237-8

[23] Harris CC . The carcinogenicity of anticancer drugs: a hazard in man. Cancer 1976; 37: 1014–1023. 766951 10.1002/1097-0142(197602)37:2+<1014::aid-cncr2820370805>3.0.co;2-z

[24] Chu EH . Induction and analysis of gene mutations in cultured mammalian somatic cells. Genetics (Austin) 1974; 78: 115–132. 4531409 10.1093/genetics/78.1.115 PMC1213171

[25] Reznikoff CA Bertram JS Brankow DW , et al. Quantitative and qualitative studies of chemical transformation of cloned C3H mouse embryo cells sensitive to postconfluence inhibition of cell division. Cancer Res 1973; 33: 3239–3249. 4796800

[26] Benedict WF Banerjee A Gardner A , et al. Induction of morphological transformation in mouse C3H/10T1/2 clone 8 cells and chromosomal damage in hamster A(T1)C1-3 cells by cancer chemotherapeutic agents. Cancer Res 1977; 37: 2202. 67887

[27] Constantinides PG Jones PA Gevers W . Functional striated muscle cells from non-myoblast precursors following 5-azacytidine treatment. Nature 1977; 267: 364–366. 68440 10.1038/267364a0

[28] Christman JK . 5-Azacytidine and 5-aza-2′-deoxycytidine as inhibitors of DNA methylation: mechanistic studies and their implications for cancer therapy. Oncogene 2002; 21: 5483–5495. 12154409 10.1038/sj.onc.1205699

[29] Jones PA Taylor SM Mohandas T , et al. Cell cycle-specific reactivation of an inactive X-chromosome locus by 5-azadeoxycytidine. Proc Natl Acad Sci U S A 1982; 79: 1215–1219. 6175964 10.1073/pnas.79.4.1215 PMC345932

[30] Koshy M Dorn L Bressler L , et al. 2-Deoxy 5-azacytidine and fetal hemoglobin induction in sickle cell anemia. Blood 2000; 96: 2379–2384. 11001887

[31] Ley TJ DeSimone J Anagnou NP , et al. 5-Azacytidine selectively increases γ-globin synthesis in a patient with β+ thalassemia. N Engl J Med 1982; 307: 1469–1475. 6183586 10.1056/NEJM198212093072401

[32] Pinto A Attadia V Fusco A , et al. 5-Aza-2′-deoxycytidine induces terminal differentiation of leukemic blasts from patients with acute myeloid leukemias. Blood 1984; 64: 922–929. 6206904

[33] Rivard GE Momparler RL Demers J , et al. Phase I study on 5-aza-2′-deoxycytidine in children with acute leukemia. Leuk Res 1981; 5: 453–462. 6173545 10.1016/0145-2126(81)90116-8

[34] Freireich EJ Gehan EA Rall DP , et al. Quantitative comparison of toxicity of anticancer agents in mouse, rat, hamster, dog, monkey, and man. Cancer Chemother Rep 1966; 50: 219–244. 4957125

[35] Schein PS . Preclinical toxicology of anticancer agents. Cancer Res 1977; 37: 1934–1937. 404039

[36] Momparler RL Frith CH . Toxicology in mice of the antileukemic agent 5-aza-2′-deoxycytidine. Drug Chem Toxicol 1981; 4: 373–381. 6178576 10.3109/01480548109017828

[37] Momparler RL Rivard GE Gyger M . Clinical trial on 5-aza-2′-deoxycytidine in patients with acute leukemia. Pharmacol Ther 1985; 30: 277–286. 2433702 10.1016/0163-7258(85)90052-x

[38] Richel DJ Colly LP Kluin-Nelemans JC , et al. The antileukaemic activity of 5-aza-2 deoxycytidine (Aza-dC) in patients with relapsed and resistant leukaemia. Br J Cancer 1991; 64: 144–148. 1713050 10.1038/bjc.1991.258 PMC1977302

[39] Willemze R Archimbaud E Muus P . Preliminary results with 5-aza-2′-deoxycytidine (DAC)-containing chemotherapy in patients with relapsed or refractory acute leukemia. the EORTC Leukemia Cooperative Group. Leukemia 1993; 7(Suppl. 1): 49–50. 7683357

[40] Willemze R Suciu S Archimbaud E , et al. A randomized phase II study on the effects of 5-aza-2′-deoxycytidine combined with either amsacrine or idarubicin in patients with relapsed acute leukemia: an EORTC Leukemia Cooperative Group phase II study (06893). Leukemia 1997; 11(Suppl. 1): S24–S27. 9130688

[41] Petti MC Mandelli F Zagonel V , et al. Pilot study of 5-aza-2′-deoxycytidine (decitabine) in the treatment of poor prognosis acute myelogenous leukemia patients: preliminary results. Leukemia 1993; 7(Suppl. 1): 36–41. 7683355

[42] Schwartsmann G Fernandes MS Schaan MD , et al. Decitabine (5-aza-2′-deoxycytidine; DAC) plus daunorubicin as a first line treatment in patients with acute myeloid leukemia: preliminary observations. Leukemia 1997; 11(Suppl. 1): S28–S31. 9130689

[43] Momparler RL Bouchard J Onetto N , et al. 5-Aza-2′-deoxycytidine therapy in patients with acute leukemia inhibits DNA methylation. Leuk Res 1984; 8: 181–185. 6201685 10.1016/0145-2126(84)90141-3

[44] Zagonel V Lo Re G Marotta G , et al. 5-Aza-2′-deoxycytidine (Decitabine) induces trilineage response in unfavourable myelodysplastic syndromes. Leukemia 1993; 7(Suppl. 1): 30–35. 7683354

[45] Wijermans P Lübbert M Verhoef G , et al. Low-dose 5-aza-2′-deoxycytidine, a DNA hypomethylating agent, for the treatment of high-risk myelodysplastic syndrome: a multicenter phase II study in elderly patients. J Clin Oncol 2000; 18: 956–956. 10694544 10.1200/JCO.2000.18.5.956

[46] Saxena K Konopleva M . New treatment options for older patients with acute myeloid leukemia. Curr Treat Options Oncol 2021; 22: 39. 33743079 10.1007/s11864-021-00841-4

[47] Cortes JE Mehta P . Determination of fitness and therapeutic options in older patients with acute myeloid leukemia. Am J Hematol 2021; 96: 493–507. 33368536 10.1002/ajh.26079 PMC7986910

[48] Issa J-PJ Garcia-Manero G Giles FJ , et al. Phase 1 study of low-dose prolonged exposure schedules of the hypomethylating agent 5-aza-2′-deoxycytidine (decitabine) in hematopoietic malignancies. Blood 2004; 103: 1635–1640. 14604977 10.1182/blood-2003-03-0687

[49] Cashen AF Schiller GJ O’Donnell MR , et al. Multicenter, phase II study of decitabine for the first-line treatment of older patients with acute myeloid leukemia. J Clin Oncol 2010; 28: 556–561. 20026803 10.1200/JCO.2009.23.9178

[50] Kantarjian HM Thomas XG Dmoszynska A , et al. Multicenter, randomized, open-label, phase III trial of decitabine versus patient choice, with physician advice, of either supportive care or low-dose cytarabine for the treatment of older patients with newly diagnosed acute myeloid leukemia. J Clin Oncol 2012; 30: 2670–2677. 22689805 10.1200/JCO.2011.38.9429 PMC4874148

[51] Ritchie EK Feldman EJ Christos PJ , et al. Decitabine in patients with newly diagnosed and relapsed acute myeloid leukemia. Leuk Lymphoma 2013; 54: 2003–2007. 23270581 10.3109/10428194.2012.762093 PMC3888021

[52] Blum W Garzon R Klisovic RB , et al. Clinical response and miR-29b predictive significance in older AML patients treated with a 10-day schedule of decitabine. Proc Natl Acad Sci U S A 2010; 107: 7473–7478. 20368434 10.1073/pnas.1002650107 PMC2867720

[53] DiNardo CD Maiti A Rausch CR , et al. 10-Day decitabine with venetoclax for newly diagnosed intensive chemotherapy ineligible, and relapsed or refractory acute myeloid leukaemia: a single-centre, phase 2 trial. Lancet Haematol 2020; 7: e724–e736. 10.1016/S2352-3026(20)30210-6 PMC7549397 32896301

[54] Yilmaz M Muftuoglu M Kantarjian H , et al. S127: quizartinib with decitabine and venetoclax (triplet) is active in patients with FLT3-ITD mutated acute myeloid leukemia – a phase I/II study. HemaSphere 2022; 6: 28–29.

[55] Zeidan AM Jayade S Schmier J , et al. Injectable hypomethylating agents for management of myelodysplastic syndromes: patients’ perspectives on treatment. Clin Lymphoma, Myeloma Leuk 2022; 22: e185–e198. 10.1016/j.clml.2021.09.009 34674983

[56] Mistry B Jones MM Kubiak P , et al. A phase 1 study to assess the absolute bioavailability and safety of an oral solution of decitabine in subjects with myelodysplastic syndromes (MDS). Blood 2011; 118: 3801–3801.

[57] Camiener GW Smith CG . Studies of the enzymatic deamination of cytosine arabinoside. I. Enzyme distribution and species specificity. Biochem Pharmacol 1965; 14: 1405–1416. 4956026 10.1016/0006-2952(65)90175-9

[58] Savona MR Odenike O Amrein PC , et al. An oral fixed-dose combination of decitabine and cedazuridine in myelodysplastic syndromes: a multicentre, open-label, dose-escalation, phase 1 study. Lancet Haematol 2019; 6: e194–e203. 10.1016/S2352-3026(19)30030-4 30926081

[59] Garcia-Manero G Griffiths EA Steensma DP , et al. Oral cedazuridine/decitabine for MDS and CMML: a phase 2 pharmacokinetic/pharmacodynamic randomized crossover study. Blood 2020; 136: 674–683. 32285126 10.1182/blood.2019004143 PMC7414597

[60] Tsujimoto Y Finger LR Yunis J , et al. Cloning of the chromosome breakpoint of neoplastic B cells with the t(14;18) chromosome translocation. Science 1984; 226: 1097–1099. 6093263 10.1126/science.6093263

[61] Vaux DL . Early work on the function of Bcl-2, an interview with David Vaux. Cell Death Diff 2004; 11: S28–S32. 10.1038/sj.cdd.4401439 15118765

[62] Hockenbery DM Zutter M Hickey W , et al. BCL2 protein is topographically restricted in tissues characterized by apoptotic cell death. Proc Natl Acad Sci U S A 1991; 88: 6961–6965. 1871110 10.1073/pnas.88.16.6961 PMC52213

[63] Bogenberger JM Delman D Hansen N , et al. Ex vivo activity of BCL-2 family inhibitors ABT-199 and ABT-737 combined with 5-azacytidine in myeloid malignancies. Leuk Lymphoma 2015; 56: 226V229. 10.3109/10428194.2014.910657 PMC4331188 24707940

[64] DiNardo CD Pratz K Pullarkat V , et al. Venetoclax combined with decitabine or azacitidine in treatment-naive, elderly patients with acute myeloid leukemia. Blood 2019; 133: 7–17. 30361262 10.1182/blood-2018-08-868752 PMC6318429

[65] DiNardo CD Jonas BA Pullarkat V , et al. Azacitidine and venetoclax in previously untreated acute myeloid leukemia. N Engl J Med 2020; 383: 617–629. 32786187 10.1056/NEJMoa2012971

[66] Chua CC Hammond D Kent A , et al. Treatment-free remission after ceasing venetoclax-based therapy in patients with acute myeloid leukemia. Blood Adv 2022; 6: 3879–3883. 35511730 10.1182/bloodadvances.2022007083 PMC9278306

[67] Almanza-Huante E Bataller A Urrutia S , et al. Outcomes of patients with therapy-related myeloid neoplasms after treatment with poly(ADP-ribose) polymerase proteins inhibitors for solid tumours. Br J Haematol 2023; 201: e25–e29. 10.1111/bjh.18766 36951293

[68] Morice PM Leary A Dolladille C , et al. Myelodysplastic syndrome and acute myeloid leukaemia in patients treated with PARP inhibitors: a safety meta-analysis of randomised controlled trials and a retrospective study of the WHO pharmacovigilance database. Lancet Haematol 2021; 8: e122–e134. 10.1016/S2352-3026(20)30360-4 33347814

[69] Kim K Maiti A Loghavi S , et al. Outcomes of TP53-mutant acute myeloid leukemia with decitabine and venetoclax. Cancer 2021; 127: 3772–3781. 34255353 10.1002/cncr.33689 PMC10462434

[70] Bernard E Nannya Y Hasserjian RP , et al. Implications of TP53 allelic state for genome stability, clinical presentation and outcomes in myelodysplastic syndromes. Nat Med 2020; 26: 1549–1556. 32747829 10.1038/s41591-020-1008-z PMC8381722

